# Disturbed balance of expression between XIAP and Smac/DIABLO during tumour progression in renal cell carcinomas

**DOI:** 10.1038/sj.bjc.6602127

**Published:** 2004-08-24

**Authors:** Y Yan, C Mahotka, S Heikaus, T Shibata, N Wethkamp, J Liebmann, C V Suschek, Y Guo, H E Gabbert, C D Gerharz, U Ramp

**Affiliations:** 1 Institute of Pathology, University Hospital, Duesseldorf, Germany; 2 Institute of Molecular Medicine, University Hospital, Duesseldorf, Germany; 3 Institute of Urology, First Hospital, Peking University, China

**Keywords:** X-linked inhibitor of apoptosis, second mitochondria-derived activator of caspases, direct IAP-binding protein with low pI, renal cell carcinoma, tumour progression

## Abstract

Dysregulation of apoptosis plays an important role in tumour progression and resistance to chemotherapy. The X-linked inhibitor of apoptosis (XIAP) is considered to be the most potent caspase inhibitor of all known inhibitor of apoptosis-family members. Only recently, an antagonist of XIAP has been identified, termed Smac/DIABLO. To explore the relevance of antiapoptotic XIAP and proapoptotic Smac/DIABLO for tumour progression in renal cell carcinomas (RCCs), we analysed XIAP and Smac/DIABLO mRNA and protein expression in the primary tumour tissue from 66 RCCs of all major histological types by quantitative real-time PCR, Western blot and ELISA. X-linked inhibitor of apoptosis and Smac/DIABLO mRNA expression was found in all RCCs. Importantly, the relative XIAP mRNA expression levels significantly increased from early (pT1) to advanced (pT3) tumour stages (*P*=0.0002) and also with tumour dedifferentiation (*P*=0.04). Western blot analysis confirmed the tumour stage-dependent increase of XIAP expression on the protein level. In contrast, mRNA and protein expression levels of Smac/DIABLO did not significantly change between early and advanced tumour stages or between low and high tumour grades. Consequently, the mRNA expression ratio between antiapoptotic XIAP and proapoptotic Smac/DIABLO markedly increased during progression from early (pT1) to advanced (pT3) tumour stages. Moreover, RCCs confined within the organ capsule (pT1 and pT2) exhibited a significantly lower XIAP to Smac/DIABLO expression ratio when compared with RCCs infiltrating beyond the kidney (pT3; *P*=0.01). Thus, our investigation demonstrates that the delicate balance between XIAP and Smac/DIABLO expression is gradually disturbed during progression of RCCs, resulting in a relative increase of antiapoptotic XIAP over proapoptotic Smac/DIABLO, thereby probably contributing to the marked apoptosis resistance of RCC.

Resistance to apoptosis plays a critical role during tumorigenesis and tumour progression, facilitating the accumulation of transforming mutations, promoting evasion of tumour cells from immunosurveillance and contributing to resistance against anticancer drugs (Gerharz *et al*, 1999; Reed, 1999; Ramp *et al*, 2000, 2003a, 2003b; Igney and Krammer, 2002). The molecular pathways of apoptotic cell death are controlled by genes that either promote or inhibit the activation of the caspase cascade and, consequently, an imbalance between proapoptotic and antiapoptotic regulators may significantly contribute to apoptosis resistance.

Recently, the activity of caspases has been shown to be inhibited by members of the inhibitor of apoptosis protein (IAP) family (Salvesen and Duckett, 2002). Up to now, eight human IAP proteins have been identified: X-linked inhibitor of apoptosis (XIAP), survivin, c-IAP1, c-IAP2, NAIP, BRUCE, ILP-2 and ML-IAP (Livin) (Mahotka *et al*, 1999, 2002a, 2002b; Reed, 1999; Salvesen and Duckett, 2002). X-linked inhibitor of apoptosis has been shown to be the most potent caspase-inhibitory IAP family member that is able to inhibit caspase-3, -7 and -9 (Deveraux *et al*, 1999; Huang *et al*, 2001; Srinivasula *et al*, 2001). Moreover, XIAP exhibits E3 ligase activity and is able to ubiquitinate caspase-3 for degradation by the proteasome (Suzuki *et al*, 2001). X-linked inhibitor of apoptosis, therefore, acts as a potent suicide inactivator, effectively inhibiting apoptosis induced by a variety of extracellular stimuli, including anticancer drugs and ionising radiation (Li *et al*, 2000; Asselin *et al*, 2001; Holcik *et al*, 2001; Matsumiya *et al*, 2001).

Only recently, three inhibitors of XIAP have been identified, namely Second Mitochondria-derived Activator of Caspases/Direct IAP-Binding Protein with Low PI (Smac/DIABLO), Omi/HtrA2 (Hedge *et al*, 2002; Verhagen *et al*, 2002; Martins *et al*, 2003; Srinivasula *et al*, 2003) and XIAP-Associated Factor 1 (XAF1) (Fong *et al*, 2000; Liston *et al*, 2001; Verhagen and Vaux, 2002). The mechanism by which XAF1 antagonises the effects of XIAP is not very well known, but might affect the redistribution of XIAP from the cytoplasm to the nucleus (Liston *et al*, 2001). Omi/HtrA2 and Smac/DIABLO are localised within the mitochondria and are released into the cytosol upon apoptotic stimuli. Cytosolic Omi/HtrA2 results in displacement of XIAP from caspases and loss of their suppressive effect on caspase activity (Hedge *et al*, 2002; Verhagen *et al*, 2002; Martins *et al*, 2003). Moreover, Omi/HtrA2 was shown to be able to degrade XIAP protein (Martins *et al*, 2003; Srinivasula *et al*, 2003). Cytosolic and proteolytically processed Smac/DIABLO can also disrupt the interaction between XIAP and caspases, thereby relieving the inhibition of caspase-3, -7 and -9 by XIAP (Srinivasula *et al*, 2001; van Loo *et al*, 2002; Verhagen and Vaux, 2002). Since overexpression of Smac/DIABLO was able to sensitise tumour cells against anticancer drug- and TRAIL-induced apoptosis (Zhang *et al*, 2001; Guo *et al*, 2002; Ng *et al*, 2002; Mc Neish *et al*, 2003), Smac/DIABLO is thought to be a major antagonist of XIAP.

Importantly, inhibition of apoptosis by IAPs also seems to promote tumour progression *in vivo*, suggesting that IAPs might be used as novel prognostic factors. Thus, AML patients with low XIAP levels exhibited a significantly longer survival time (Tamm *et al*, 2000). Conflicting observations, however, have been reported between XIAP expression and tumour stage or patient's survival in carcinomas of the cervix as well as non-small-cell lung carcinomas of early or advanced stages (Ferreira *et al*, 2001a, 2001b; Liu *et al*, 2001; Hofmann *et al*, 2002). Preliminary investigations demonstrated expression of Smac/DIABLO in 62% of various carcinoma types (Yoo *et al*, 2003), but the relation between Smac/DIABLO expression and tumour staging, grading or survival has not been analysed yet. Moreover, since XIAP and Smac/DIABLO are major opponents, it is reasonable to assume that the expression of both – rather than the expression of a single regulator – may be relevant for regulating apoptosis susceptibility.

The aim of the present investigation, therefore, was to explore the relevance of XIAP and Smac/DIABLO expression for tumour progression in a large cohort of renal cell carcinomas (RCCs) of all major histological types.

## MATERIAL AND METHODS

### Patients and specimens

Tumour tissue samples of 66 consecutive RCCs of different histological types, grades and stages (Table 1

**Table 1:** Pathological staging, grading and typing of renal call carcinomas

	**Staging**		**Grading**		**Typing**	
XIAP (*n*=59)	pT1	25	G1	2	Clear cell	46
	pT2	20	G2	44	Papillary	11
	pT3	14	G3	13	Chromophob	2
						
Smac/DIABLO (*n*=66)	pT1	22	G1	1	Clear cell	56
	pT2	21	G2	52	Papillary	9
	pT3	23	G3	13	Chromophob	1
						
XIAP/Smac (*n*=46)	pT1	16	G1	1	Clear cell	37
	pT2	17	G2	34	Papillary	8
	pT3	13	G3	11	Chromophob	1

) were obtained from patients who had undergone nephrectomy. The tumour specimens were immediately flash frozen in liquid nitrogen and stored at −80°C until RNA extraction. Tumour typing, grading and staging were performed according to the principles outlined by the WHO (Mostofi and Davis, 1998), Störkel *et al* (1989) and the UICC (Wittekind and Wagner, 1997). The proportion of the different RCC types in our study reflects the frequency of each tumour type in a large RCC series (Mostofi and Davis, 1998).

### RNA extraction

Total RNA was isolated by the guanidium thiocyanate extraction method as described by Chomczynski and Sacchi (1987). Afterwards, the total RNA was purified using the RNeasy minikit with DNAse treatment according to the manufacturer's instructions (Qiagen, Hilden, Germany) to exclude DNA contamination. The integrity of all tested total RNA samples was verified by intact 18S/28S rRNA bands in agarose gel electrophoresis.

### Reverse transcription

For cDNA synthesis, 1 *μ*g of total RNA was reversed transcribed in a final volume of 20 *μ*l containing 15 *μ*M of each dNTP (Roche, Germany), 10 U of recombinant RNasin RNase inhibitor (Promega, Heidelberg, Germany) and 2.5 U of AMV reverse transcriptase (Promega, Heidelberg, Germany) with the corresponding 1 × RT buffer. The following sets of primers were used: an XIAP-specific oligonucleotide primer (10 pmol; 5′-TTC CTC GGG TAT ATG GTG TCT GAT-3′) or a Smac/DIABLO-specific primer (10 pmol; 5′-TTC AAT CAA CGC ATA TGT GGT CTG-3′) that specifically hybridise at the 3′ regions of XIAP and Smac/DIABLO, respectively. The sequence-specific antisense GAPDH primer was: 5′-TGT CAT CAT ATT TGG CAG GTT T-3′ (10 pmol). Reverse transcriptions (RTs) of both of XIAP and GAPDH as well as both of Smac/DIABLO and GAPDH were performed in the same reactions to avoid variations in the efficiency of cDNA synthesis. The RT reaction mixtures were incubated at 55°C for 1 h.

### Quantitative real-time detection PCR

Amplification and quantification of XIAP, Smac/DIABLO and GAPDH were carried out in the LightCycler (Roche Diagnostics, Mannheim, Germany) by using a total volume of 20 *μ*l, including 2 *μ*l 10 × SYBR Green I Fast Start Reaction Mix (Roche Diagnostics, Mannheim, Germany), 3 mM MgCl_2_, 25 pmol of each upstream and downstream XIAP-specific oligonucleotide primers (forward primer: 5′-CCG TGC GGT GCT TTA GTT GT-3′; reverse primer: 5′-TTC CTC GGG TAT ATG GTG TCT GAT-3′) (GeneBank accession number U45880), Smac/DIABLO-specific oligonucleotide primers (forward primer: 5′-TGT GAC GAT TGG CTT TGG AGT AAC-3′; reverse primer: 5′-TTC AAT CAA CGC ATA TGT GGT CTG-3′) and GAPDH-specific oligonucleotide primers (forward primer: 5′-TTG GTA TCG TGG AAG GAC TCA-3′; reverse primer: 5′-TGT CAT CAT ATT TGG CAG GTT T-3′), 2 *μ*l of first-strand cDNA template from RT (or water as negative control). Real-time PCR was carried out in glass capillaries with an initial denaturation step of 10 min at 95°C, followed by 50 cycles of 0 s at 95°C, annealing for 10 s at 61°C and elongation for 10 s at 72°C. Melting curve was directly drawn after amplification.

PCR products were additionally checked by electrophoresis on 3% agarose gels containing ethidium bromide and visualised under UV transillumination. PCR products were confirmed by DNA sequencing. Briefly, products were excised from agarose gels and isolated by QIAquick gel extraction kit (Qiagen, Hilden, Germany). The purified PCR products were sequenced using the ABI-Prism BigDye Terminator Cycle Sequencing kit (ABI, Weiterstadt, Germany) with the respective forward and reverse primers used in the real-time PCR according to the manufacturer's protocol. Sequence analysis was carried out using an ABI-Prism 310.

### Quantification of XIAP and Smac/DIABLO by an external standard

A cDNA fragment of the housekeeping gene GAPDH was cloned. Plasmids (pGEM-T-easy; Promega, Heidelberg, Germany) were isolated, linearised by *Sac*I restriction and cleaned. Plasmid concentration was measured and the number of GAPDH copies was calculated. Serial dilutions of the linearised GAPDH plasmid from 6 × 10^8^–3 × 10^6^ copies were prepared. For relative quantification of XIAP, Smac/DIABLO and GAPDH copy numbers, a standard curve was created by plotting the crossing point (CP) values against the number of copies. Every dilution was run at least in duplicate. The number of gene copies was calculated by the LightCycler Software (Version 3.5) according to the second derivative maximum method.

### Western blot analysis

Protein extracts from 12 arbitrarily selected pT1 and pT3 RCCs were isolated by disrupting flash frozen tissue samples in lysis buffer (100 mM NaCl, 10 mM Tris–HCl, pH 7.6, 1 mM EDTA, pH 8.0, 1% NP40 and protease inhibitors). Protein aliquots (up to 50 *μ*g) were electrophoresed in 12% SDS–polyacrylamide gels at 70 mA for 4 h. Blotting to Opitran BA-S85 nitrocellulose membranes (Schleicher & Schuell, Dassel, Germany) was performed for up to 2 h at 650 mA in a tank of transfer buffer (25 mM Tris–HCl, 192 mM glycine, 20% methanol; pH 8.3), using the Hoefer TE series Transphor Electrophoresis Unit (Hoefer Scientific Instruments, San Francisco, USA). To verify transfer efficiency, nitrocellulose membranes were stained with 0.2% Ponceau S. The membranes were blocked for 2 h at 4°C in blocking buffer (100 mM Tris–HCl, pH 7.5, 150 mM NaCl, 0.2% Tween 20) plus 3% non-fat dry milk and 1% BSA, incubated overnight at 4°C with the polyclonal rabbit anti-human XIAP antibody (No. 2042, Cell Signaling Technology, England) by dilution of 1 : 500, and incubated after washing with the horseradish peroxidase-linked donkey anti-rabbit antibody for 1 h at room temperature by 1 : 2000 dilution. After washing, the protein was visualised by incubation with Lumi-Light substrate (Roche, Mannheim, Germany). Equal amounts of the loaded protein samples were confirmed by *β*-actin detection with the mouse monoclonal anti-human *β*-actin antibody (clone AC-15; Sigma-Aldrich, Deisenhofen, Germany).

### Enzyme-linked immunosorbent assay (ELISA)

For quantification of Smac/DIABLO protein expression in RCCs, quantitative sandwich ELISA was carried out using the DuoSet® IC assay kit (R&D Systems, Wiesbaden, Germany) as described by the manufacturer's manual. Briefly, proteins were isolated from 24 arbitrarily selected clear cell RCCs (pT1: *n*=8, pT2: *n*=8, pT3: *n*=8) and diluted to a concentration of 400 *μ*g ml^−1^. A standard curve was generated by using two-fold serial dilutions and a high standard of 5000 pg ml^−1^. A 96-well microtitre plate was coated overnight at room temperature by capture antibody and, after washing with washing buffer (0.05% Tween 20 in PBS, pH 7.3), blocked with blocking buffer (1% BSA, 5% sucrose in PBS, pH 7.3, with 0.05% NaN_3_) for 2 h, followed by washing. Samples or standards of 100 *μ*l were added in duplicate and incubated for 2 h. After washing, 100 *μ*l of biotinylated rabbit anti-human Smac/DIABLO antibody (R&D Systems, Wiesbaden, Germany; concentration 150 ng ml^−1^) was added to each well and incubated for 2 h. After washing again, 100 *μ*l of streptavidin conjugated to horseradish peroxidase (1 : 200 dilution) was added and incubated for 20 min, followed by washing. In all, 100 *μ*l of substrate solution (1 : 1 mixture of H_2_O_2_ and tetramethylbenzidine) was added and incubated for 20 min, and then stopped by 2 N H_2_SO_4_. Optical density was determined immediately at 450 and 570 nm in a spectrophotometer and the values at 570 nm were subtracted from the values at 450 nm. The absorbances after subtraction were directly used for statistical analysis.

### Statistical analysis

GAPDH copy numbers (external standard) were used to normalise the copy numbers of XIAP and Smac/DIABLO, calculating ratios of relative mRNA levels of XIAP, Smac/DIABLO and XIAP to Smac/DIABLO. Statistical analysis was performed using the Mann–Whitney and Wilcoxon tests with the statistical software package SPSS 11.0 (SPSS, Chicago, USA). A *P*-value of less than 0.05 was considered to indicate the statistical significance.

## RESULTS

### Quantification of XIAP and Smac/DIABLO expression

**Figure 1:**
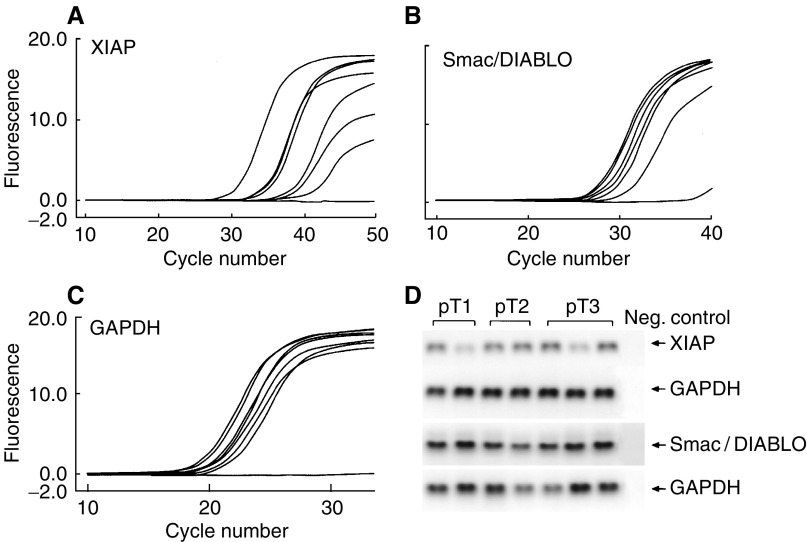
Analysis of XIAP and Smac/DIABLO mRNA expression in renal cell carcinomas by real-time detection PCR and agarose gel electrophoresis. (**A**–**C**) SYBR green I-mediated fluorescence (*y*-axis) of the respective amplification products was measured once per cycle (*x*-axis). X-linked inhibitor of apoptosis (**A**), Smac/DIABLO (**B**) and GAPDH (**C**) were detectable at cycles 19–42, 24–38 and 17–26, respectively. Comparative analysis of GAPDH transcripts was used as reference. (**D**) The specificity of the corresponding XIAP, Smac/DIABLO and GAPDH amplification products from RCCs of different stages were demonstrated by agarose gel electrophoresis, showing specific products at the calculated sizes at the end point of LightCycler PCR.

**Figure 2:**
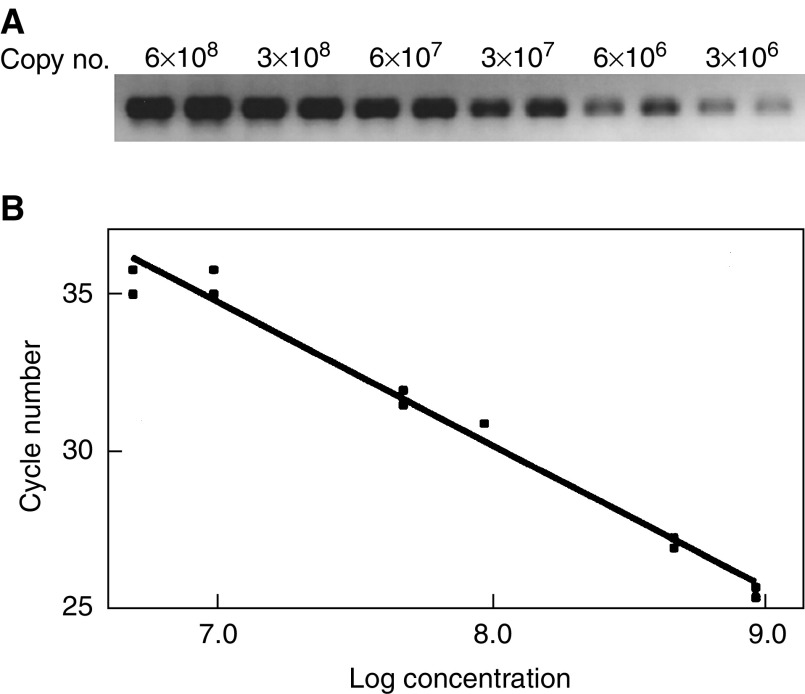
GAPDH standard curve. (**A**) Calibration of GAPDH standard by 6 × 10^8^–3 × 10^6^ copies in duplicate visualised on agarose gel. (**B**) LightCycler-based standard curve report for serially diluted GAPDH plasmid amplification. The cycle number was plotted against the log of concentration by the second derivative maximum method. A correlation coefficient of *r*=0.99 indicates a precise log-linear relationship.

X-linked inhibitor of apoptosis, Smac/DIABLO and the house-keeping gene GAPDH could be detected at cycles 19–42, 24–38 and 17–26, respectively (Figure 1A–C

). The specificity of the amplification products was confirmed by agarose gel electrophoresis, which revealed distinct bands for all PCR products (Figure 1d) and by DNA sequencing (data not shown). Cloning of a GAPDH cDNA fragment permitted the generation of a standard curve by serial dilution (Figure 2

). The standard curve with a correlation coefficient of 0.99 was used for a relative quantification of the copy numbers obtained from XIAP, Smac/DIABLO and GAPDH in each tumour sample. GAPDH copy numbers were used as an external standard to normalise the copy numbers of XIAP and Smac/DIABLO, calculating the relative mRNA levels (i.e. GAPDH-normalised mRNA expression level).

### Tumour stage- and grade-dependent increase of XIAP expression

mRNA expression of XIAP was found in all RCCs (*n*=59), irrespective of their tumour stages, grades or histological types.

**Figure 3:**
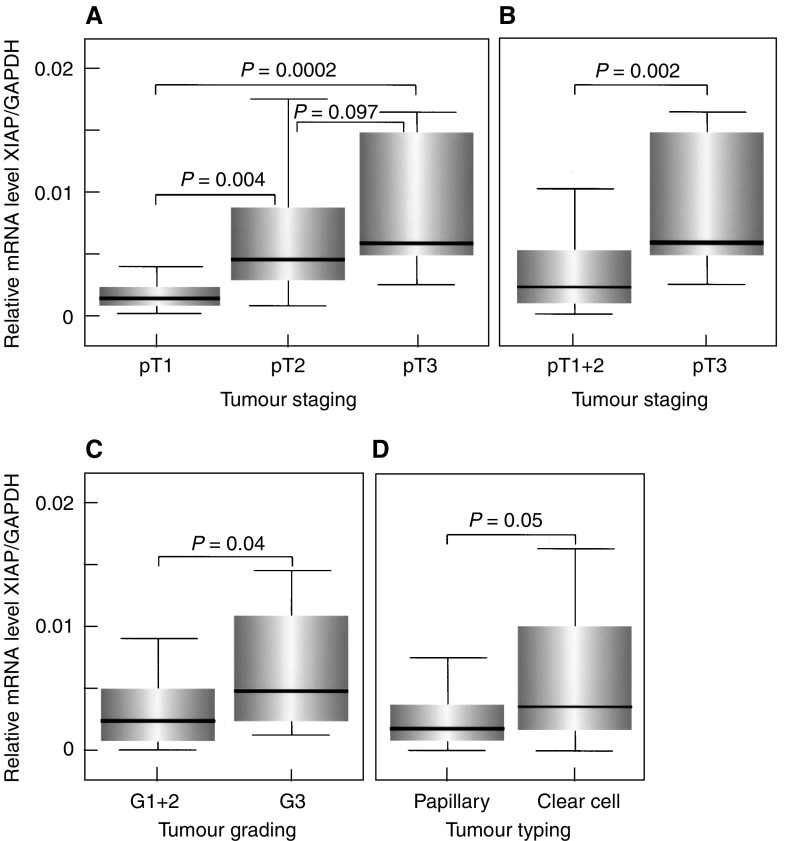
Analysis of GAPDH-normalised XIAP mRNA expression in RCCs. (**A**) Significant increase of antiapoptotic XIAP mRNA expression from early (pT1) to advanced tumour stages (pT3). (**B**) Significantly higher XIAP mRNA levels in RCCs infiltrating beyond the kidney (pT3) when compared with RCCs confined to the organ (pT1+pT2). (**C**) Significant increase in XIAP levels with dedifferentiation of RCCs. (**D**) Tendency for higher XIAP expression levels in clear cell RCCs when compared with the papillary type.

As can be seen in Figure 3A and B

, the relative mRNA expression levels of XIAP significantly increased from early (pT1) to advanced tumour stages (pT3). Importantly, RCCs infiltrating beyond the kidney (pT3) exhibited significantly higher relative XIAP mRNA expression levels when compared with tumours confined to the organ (pT1 and pT2; *P*=0.002; Figure 3A and B). Quite similarly, XIAP mRNA expression significantly increased with tumour dedifferentiation (Figure 3C). Thus, the relative expression level of XIAP was significantly higher in poorly differentiated RCCs (G3) than in well and moderately differentiated tumours (G1 and G2; *P*=0.04).

Moreover, RCCs of the clear cell type, which were reported to exhibit a poorer prognosis than papillary RCCs (Cheville *et al*, 2003) showed higher XIAP expression levels when compared with XIAP levels in papillary RCCs (Figure 3D), although the difference was of borderline significance only (*P*=0.05).

**Figure 4:**
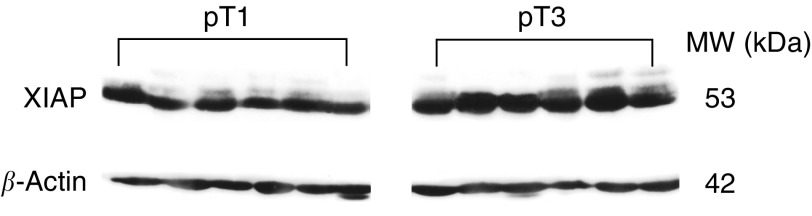
Western blot analysis of XIAP expression in 12 arbitrarily selected clear-cell RCCs. Higher XIAP protein expression in advanced RCCs (pT3) when compared with early tumour stage (pT1). Equal amounts of loaded proteins were confirmed by re-incubation of the filters with an antibody against *β*-actin.

Since XIAP expression is known to be regulated transcriptionally and post-transcriptionally (Tamm *et al*, 2000; Holcik *et al*, 2001; Hofmann *et al*, 2002), we also performed Western blot analysis in 12 arbitrarily selected RCC tumour samples of early and advanced tumour stages (pT1: *n*=6, pT3: *n*=6). These investigations revealed a distinct XIAP signal of the expected size (53 kDa) in all RCCs (Figure 4

). Remarkably, a stage-dependent increase of XIAP expression became evident also on the protein level (Figure 4), thereby further confirming our results on increased XIAP mRNA expression in advanced tumour stages.

Our results, therefore, demonstrate an increase in antiapoptotic XIAP mRNA and protein expression during tumour progression.

### Constant expression of Smac/DIABLO irrespective of tumour stage and grade

Since Smac/DIABLO is known to be a major antagonist of XIAP (van Loo *et al*, 2002; Verhagen and Vaux 2002), we also analysed the stage- and grade-dependent expression of Smac/DIABLO in RCCs.

mRNA expression of Smac/DIABLO was also found in all RCCs (*n*=66), irrespective of their staging, grading or histological typing.

**Figure 5:**
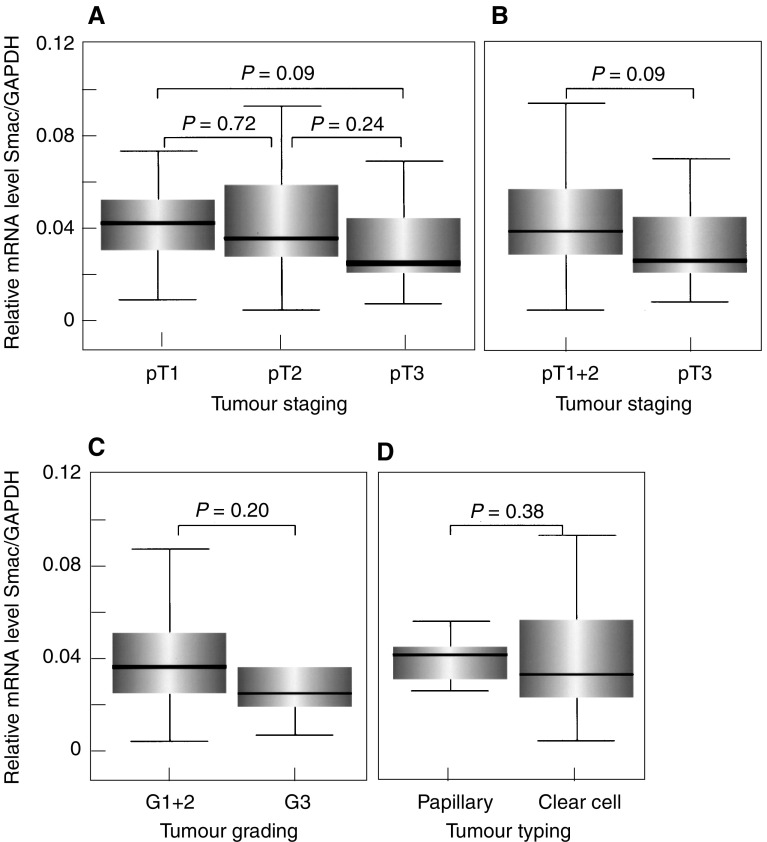
Analysis of GAPDH-normalised Smac/DIABLO mRNA expression in RCCs. (**A**, **B**) No significant changes of Smac/DIABLO mRNA expression from early (pT1) to advanced (pT3) tumour stages and between RCCs infiltrating beyond the kidney (pT3) and confined to the organ (pT1+pT2). (**C**, **D**) No significant differences between low (G1+G2) and high tumour grades (G3) or between clear cell and papillary tumour types.

Importantly, as shown in Figure 5A–D

, the relative mRNA expression levels of proapoptotic Smac/DIABLO did not significantly change between early and advanced tumour stages (Figure 5A and B) or between low and high tumour grades (Figure 5C). Similar Smac/DIABLO mRNA levels were observed in RCCs of the clear cell and papillary types (Figure 5D).

**Figure 6:**
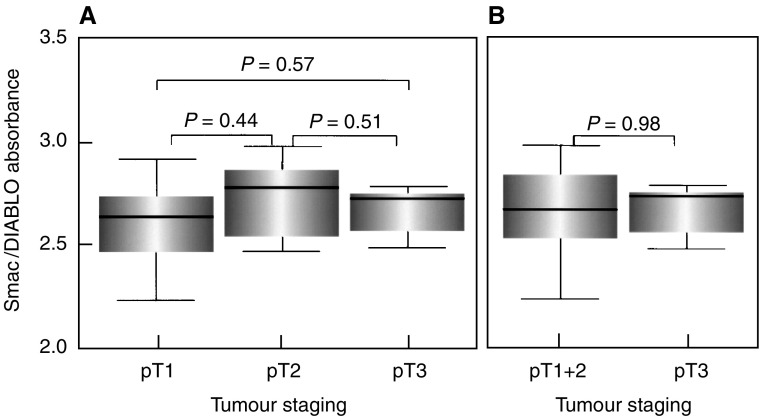
Analysis of Smac/DIABLO protein expression by quantitative ELISA in 24 arbitrarily selected RCCs. (**A**, **B**) No significant differences in Smac/DIABLO protein expression between early (pT1) and advanced (pT3) tumour stages or between RCCs confined to the organ (pT1+pT2) and infiltrating beyond the kidney (pT3).

To analyse whether Smac/DIABLO expression remains constant also at the protein level, we investigated the corresponding protein expression in 24 arbitrarily selected RCCs of different tumour stages by ELISA. As can be seen in Figure 6

, Smac/DIABLO protein levels showed no significant differences between different tumour stages.

Our results, therefore, demonstrate that expression of Smac/DIABLO mRNA and protein remains constant during RCC progression.

### Increased expression ratio of XIAP to Smac/DIABLO during tumour progression

Recent observations demonstrated an inverse expression pattern of XIAP and Smac/DIABLO after TRAIL-, UV-B-, and drug-induced apoptosis (Chow *et al*, 2003; Griffin *et al*, 2003; Takasawa and Tanuma, 2003), suggesting that the relative ratio between XIAP and Smac/DIABLO is crucial to determine apoptosis susceptibility.

**Figure 7:**
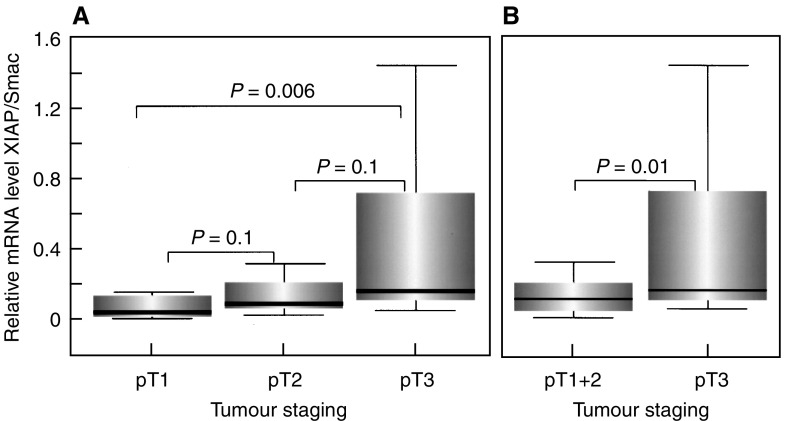
Ratio of antiapoptotic XIAP to proapoptotic Smac/DIABLO mRNA expression in different tumour stages. (**A**) Significant increase in the expression ratio between XIAP and Smac/DIABLO, comparing early (pT1) and advanced (pT3) tumour stages and (**B**) of RCCs confined to the organ (pT1+pT2) with RCCs infiltrating beyond the kidney (pT3).

We, therefore, calculated the ratio between antiapoptotic XIAP and proapoptotic Smac/DIABLO mRNA expression levels in different tumour stages. As demonstrated in Figure 7

, the mRNA expression ratio between XIAP and Smac/DIABLO markedly increased during progression from early (pT1) to advanced (pT3) tumour stages. Importantly, RCCs confined to the organ capsule (pT1 and pT2) exhibited a significantly lower expression ratio between XIAP and Smac/DIABLO when compared with advanced RCCs infiltrating beyond the kidney (pT3; *P*=0.01).

Thus, our investigation demonstrates that the delicate balance between XIAP and Smac/DIABLO expression is gradually disturbed during progression of RCCs, resulting in a relative increase of antiapoptotic XIAP over proapoptotic Smac/DIABLO.

## DISCUSSION

The present investigation demonstrates for the first time a stage- and grade-dependent increase of antiapoptotic XIAP expression in human RCCs. In contrast, the mRNA and protein levels of its antagonist Smac/DIABLO remained constant, resulting in an increased expression ratio between XIAP and Smac/DIABLO during tumour progression.

Suppression of apoptosis promotes tumour progression, immune evasion of neoplastic cells as well as resistance to chemotherapy and irradation (Gerharz *et al*, 1999; Mahotka *et al*, 1999; Reed, 1999; Ramp *et al*, 2000, 2003a, 2003b; Igney and Krammer 2002; Mahotka *et al*, 2002a, 2002b). Several genes critical in the regulation of apoptosis have been identified, including XIAP – a member of the IAP family. X-linked inhibitor of apoptosis is thought to act as a key determinant of apoptosis resistance by effectively inhibiting the activation of caspase-3, -7 and -9 (Deveraux *et al*, 1999; Holcik *et al*, 2001; Srinivasula *et al*, 2001; Suzuki *et al*, 2001). Thus, high expression of XIAP has been reported in many malignant tumour types, such as carcinomas of the breast, ovaries, lung, pancreas, cervix and prostate (Ferreira *et al*, 2001a, 2001b; Liu *et al*, 2001; Gerhard *et al*, 2002; Hofmann *et al*, 2002; Mc Eleny *et al*, 2002; Parton *et al*, 2002; Sui *et al*, 2002) as well as leukaemias (Tamm *et al*, 2000). Moreover, upregulation of XIAP expression has been found to result in apoptosis resistance after exposure to anticancer drugs and ionising radiation (Li *et al*, 2000; Asselin *et al*, 2001; Holcik *et al*, 2001; Matsumiya *et al*, 2001), whereas downregulation of XIAP by antisense vectors increased the apoptosis sensitivity of carcinoma cell lines (Sasaki *et al*, 2000; Holcik *et al*, 2001).

In our study, we found XIAP expression in all RCCs of all major histological types. This observation further confirmed the ubiquity of XIAP expression in human cancers, as reported earlier for other tumour types (Tamm *et al*, 2000). Importantly, we could demonstrate for the first time in RCCs that XIAP mRNA expression levels significantly increased from early (pT1) to advanced tumour stages (pT3) and – quite similarly – also with tumour dedifferentiation. Moreover, RCCs of the clear cell type, which were reported to exhibit a poorer prognosis than papillary RCCs (Cheville *et al*, 2003), showed a tendency for higher XIAP expression. Since XIAP expression is known to be also post-transcriptionally regulated, we additionally performed Western blot analysis in arbitrarily selected RCC samples of early and advanced tumour stages and could confirm the tumour stage-dependent increase of XIAP expression on the protein level. Similarly, a correlation between XIAP mRNA and protein levels was previously reported in non-small-cell lung carcinomas (Hofmann *et al*, 2002).

Since tumour stage and grade are considered as major prognostic parameters in RCC (Srigley *et al*, 1997), our results might also suggest that RCCs with a strong XIAP expression exhibit a poorer clinical outcome. The corresponding results were previously reported in AML, where patients with higher XIAP levels exhibited a significantly shorter survival time (Tamm *et al*, 2000). It is reasonable to assume, therefore, that in RCCs – and probably also in AML – increased expression of antiapoptotic XIAP contributes to reduced apoptosis susceptibility of tumour cells, thereby providing an important survival advantage during tumour progression. Conflicting observations, however, have been reported between XIAP expression and tumour stage or survival in other tumour types. In these reports, no correlation between XIAP-expression levels and tumour stage, grade or survival was observed in patients with carcinomas of the cervix, non-small-cell lung carcinomas and advanced nonresectable non-small-cell lung carcinomas treated by chemotherapy alone (Liu *et al*, 2001; Ferreira *et al*, 2001a; Hofmann *et al*, 2002), whereas high XIAP-expression levels were associated with longer survival in patients with radically resected non-small-cell lung carcinomas (Ferreira *et al*, 2001b).

Susceptibility for apoptosis is controlled by a multitude of anti- and proapoptotic regulators. Whereas antiapoptotic XIAP has been shown to be a potent caspase inhibitor (Deveraux *et al*, 1999; Holcik *et al*, 2001; Srinivasula *et al*, 2001; Suzuki *et al*, 2001), its antagonists Smac/DIABLO and Omi/HtrA2 promote apoptosis by binding to XIAP, thereby preventing them from inhibition of caspases (Verhagen *et al*, 2000, 2002; Martins *et al*, 2003). Importantly, overexpression of Smac/DIABLO sensitises tumour cells against anticancer drug- and TRAIL-induced apoptosis (Zhang *et al*, 2001; Guo *et al*, 2002; Ng *et al*, 2002; Mc Neish *et al*, 2003). Only recently, an inverse relation between XIAP expression and mitochondrial release of Smac/Diablo has been observed after apoptosis induction in colon cancer cells, lymphoma cells and keratinocytes (Chow *et al*, 2003; Takasawa and Tanuma, 2003; Tillman *et al*, 2003). Moreover, XIAP protein was shown to function as an E3 ligase in the ubiquitination of Smac/DIABLO, thereby promoting its rapid degradation (Mac Farlane *et al*, 2002). These observations indicate a tight regulation of these opponents and might also suggest that the expression ratio – rather than the expression of a single regulator – effectively controls susceptibility for apoptosis. Investigations, however, analysing the expression of XIAP and Smac/DIABLO in relation to tumour stage, grade and type are lacking up to now. In our study, the mRNA and protein expression levels of Smac/DIABLO remained stable during RCC progression and did not significantly change between early and advanced tumour stages, low and high tumour grades as well as different types of RCCs. Since XIAP expression significantly increased during tumour progression, the expression ratio between XIAP and Smac/DIABLO also markedly increased during progression from early to advanced RCC stages. Thus, the delicate balance between XIAP and Smac/DIABLO is gradually disturbed during progression of RCCs, probably resulting in an increased inability for Smac/DIABLO to antagonise the antiapoptotic effects of XIAP. Although investigations concerning the expression of Omi/HtrA2 in relation to tumour stage and grade are lacking, it is reasonable to assume that the disturbed balance between XIAP and Smac/DIABLO during progression of RCCs cannot be adjusted by Omi/HtrA2.

Importantly in this context, TNF- and folic acid-induced apoptosis in tubular epithelial cells, considered to be the cells of origin for clear cell and papillary RCC, has recently been shown to be associated with increased mRNA and protein expression of Smac/DIABLO (Justo *et al*, 2003). Carcinogenesis in tubular epithelial cells, therefore, might be associated with an inability to upregulate Smac/DIABLO expression, thereby generating an important selective growth advantage. Moreover, the inappropriate increase of antiapoptotic XIAP over proapoptotic Smac/DIABLO during progression of RCCs promotes the acquisition of apoptosis resistance, which might also contribute to the clinically known resistance of RCCs to anticancer drugs and irradation.

In conclusion, our investigation demonstrates for the first time a stage- and grade-dependent increase of antiapoptotic XIAP expression in RCCs, whereas the mRNA and protein levels of its antagonist Smac/DIABLO remained constant. As a consequence, the delicate balance between XIAP and Smac/DIABLO expression is gradually disturbed during progression of RCC, probably contributing to the marked apoptosis resistance of RCCs.
